# The COVID-19 lockdown: a curse or a blessing for acute cardiovascular disease?

**DOI:** 10.1007/s12471-021-01560-z

**Published:** 2021-03-15

**Authors:** J. I. Verhoeven, T. J. F. ten Cate, F. E. de Leeuw

**Affiliations:** 1 Department of Neurology, Radboud UMC, Donders Institute for Brain, Cognition and Behaviour, Nijmegen, The Netherlands; 2 Department of Cardiology, Radboud UMC, Nijmegen, The Netherlands

In this issue of the *Netherlands Heart Journal*, de Koning et al. [1] report a significantly reduced number of ambulance rides for chest pain and ST-elevation myocardial infarction during the first 6 weeks of the national lockdown due to the coronavirus disease 2019 (COVID-19) pandemic in the Hollands-Midden region of the Netherlands. The incidence of out-of-hospital cardiac arrest remained the same as in the same period in 2019. Others have reported similar findings. In August 2020, Solomon et al. reported an alarming decrease in the number of hospitalisations for acute myocardial infarctions in Northern California, with an incidence rate ratio of 0.52 (95% confidence interval 0.40–0.68) in April 2020, compared with January–February 2020 [2]. Moreover, similar observations came from studies that investigated the incidence of other acute cardiovascular events, such as ischaemic stroke. A small study performed in Ohio compared the number of stroke alerts in February 2020 with January 2020 and found a significant decrease in daily stroke alerts, stroke diagnoses and intravenous thrombolysis performed at the emergency department, but no difference in intra-arterial thrombectomies [3]. Similarly, a population-based study in Brazil showed a significant reduction in patients presenting at the emergency room with transient ischaemic attack and mild-to-moderate stroke, but not for severe stroke since the lockdown measures were implemented [4].

With their study, de Koning and colleagues provide novel insights compared with earlier studies. They report both fewer ambulance calls and fewer acute myocardial infarctions in hospital [1]. Unfortunately, because they used administrative data there is only limited information on medical patient characteristics and individual clinical outcomes. Also there is a risk of selection bias, since there is no information on people with potential symptoms that either do not seek any help or reach out to the general practitioner. Thus, based on these results we can only speculate on the reasons behind this remarkable decrease in the number of acute cardiovascular events. The authors consider three possible explanations: namely behavioural, pathophysiological and environmental changes during the COVID-19 pandemic.

It does seem likely that behavioural changes, such as not seeking medical help because of a fear of contracting the virus or unnecessarily burdening medical services, have influenced the observed number of acute cardiovascular events registered. Following this train of thought, it means that acute stroke or acute myocardial infarction did happen, but the patients did not seek medical care, except when medical assistance was unavoidable, such as for out-of-hospital cardiac arrests or disabling ischaemic stroke. This is supported by the fact that de Koning et al. [1] did not observe a decreased number of out-of-hospital cardiac arrests, and a previous larger study even found an increase [5]. If this were true, one would expect patients that initially did not seek medical help to present with the chronic effects of untreated acute cardiovascular events *after* the COVID-19 crisis. Whether this reservoir of untreated patients truly exists remains to be seen, but it seems unlikely that this behavioural change is the only explanation for the decreasing number of acute cardiovascular events.

Another explanation could be that there were in fact fewer acute events. This may be because there were fewer acute triggers. There is a wealth of physiological, emotional and environmental triggers that can elicit an acute cardiovascular event in a patient that is vulnerable because of, for example, pre-existing atherosclerotic plaques or a hypercoagulable state [6]. During the pandemic there are presumably fewer triggers, such as less physically strenuous activity and less work-related stress. Conversely, the lockdown has also resulted in an increase in vascular risk factors, including weight gain, a more sedentary lifestyle and arguably more stress for people that struggle to keep their business alive and to suddenly have to homeschool their children. Another noteworthy trigger that is substantially less prevalent during the global lockdown is ambient (outdoor) air pollution, which may therefore also have contributed to the observed lower incidence of acute cardiovascular events. Over the last few decades, growing attention has been given to the impact of ambient air pollution on the incidence of and mortality from cardiovascular disease. Feigin et al. [7, 8] demonstrated in 2016 that ambient air pollution contributed to 17% of the 15 million strokes occurring worldwide each year. Moreover, this ambient air pollution also contributed to 17% of deaths from ischaemic heart disease and 14% of deaths from stroke globally [9].

During the global lockdown, for the first time since the Industrial Revolution, air quality has improved significantly. A recent study showed that the concentration of air pollutant (PM_2.5_) decreased by 17% across Europe and up to 30% in China during the COVID-19 lockdown in February and March, compared with the same period in 2016–2019. This dramatic decrease is attributed to less industrial activity and also to much less traffic activity [10].

Several studies have demonstrated that short-term increased exposure to air pollutants of only a few days was associated with an increase in hospitalisations for both ischaemic strokes and ischaemic heart disease [11, 12]. Pathophysiological studies have shown that after inhalation, small pollutant particles are transported into the systemic circulation and trigger an acute inflammatory response with increased thrombogenicity and plaque vulnerability [13, 14]. It has also been hypothesised that the pollutant particles themselves increase autonomic dysregulation, resulting in a higher risk of arrhythmia, which can also result in either ischaemic stroke or acute myocardial infarction [13].

Interestingly, a recent Italian study specifically investigated the effect of these decreased air pollution levels and the decreasing number of hospitalisations for acute myocardial infarction during the current pandemic. When they corrected for COVID-19-related variables, such as lockdown measures, national number of COVID-19 tests, confirmed cases and deaths, they found a significant association between decreased levels of ambient nitrogen dioxide (NO_2_) and a decrease in hospitalisations for acute myocardial infarction [15]. The fewer ambulance calls and lower than expected number of patients with acute cardiovascular disease presenting to the hospital may therefore reflect a true decrease in acute events, at least partly explained by improved air quality.

Future studies should investigate whether the incidence of acute cardiovascular events remains lower during a period in which the fear of COVID-19 has decreased in society and we are more accustomed to the lockdown, but when air pollution levels and the prevalence of acute triggers also remain lower. It should also be studied whether the presumed reservoir of patients that did suffer an acute cardiovascular event but did not seek medical help truly exists and if they present with the sequelae when this crisis is over.

## References

[1] de Koning ER, Boogers MJ, Bosch J, de Visser M, Schalij MJ, Beeres SLMA (2021). Emergency medical service evaluations for chest pain during the COVID-19 lockdown in Hollands-Midden, the Netherlands. Neth Heart J.

[2] Solomon MD, McNulty EJ, Rana JS (2020). The Covid-19 pandemic and the incidence of acute myocardial infarction. N Engl J Med.

[3] Uchino K, Kolikonda MK, Brown D (2020). Decline in stroke presentations during COVID-19 surge. Stroke.

[4] Diegoli H, Magalhaes PSC, Martins SCO (2020). Decrease in hospital admissions for transient ischemic attack, mild, and moderate stroke during the COVID-19 era. Stroke.

[5] Baldi E, Sechi GM, Mare C (2020). Out-of-hospital cardiac arrest during the Covid-19 outbreak in Italy. N Engl J Med.

[6] Schwartz BG, Kloner RA, Naghavi M (2018). Acute and subacute triggers of cardiovascular events. Am J Cardiol.

[7] Feigin VL, Roth GA, Naghavi M (2016). Global burden of stroke and risk factors in 188 countries, during 1990–2013: a systematic analysis for the Global Burden of Disease Study 2013. Lancet Neurol.

[8] Feigin VL, Nichols E, Alam T (2019). Global, regional, and national burden of neurological disorders, 1990–2016: a systematic analysis for the Global Burden of Disease Study 2016. Lancet Neurol.

[9] Cohen AJ, Brauer M, Burnett R (2017). Estimates and 25-year trends of the global burden of disease attributable to ambient air pollution: an analysis of data from the Global Burden of Diseases Study 2015. Lancet.

[10] Giani P, Castruccio S, Anav A, Howard D, Hu W, Crippa P (2020). Short-term and long-term health impacts of air pollution reductions from COVID-19 lockdowns in China and Europe: a modelling study. Lancet Planet Health.

[11] Shah AS, Lee KK, McAllister DA (2015). Short term exposure to air pollution and stroke: systematic review and meta-analysis. BMJ.

[12] Mustafic H, Jabre P, Caussin C (2012). Main air pollutants and myocardial infarction: a systematic review and meta-analysis. JAMA.

[13] Franchini M, Mannucci PM (2011). Thrombogenicity and cardiovascular effects of ambient air pollution. Blood.

[14] Xu H, Wang T, Liu S (2019). Extreme levels of air pollution associated with changes in biomarkers of atherosclerotic plaque vulnerability and thrombogenicity in healthy adults. Circ Res.

[15] Versaci F, Gaspardone A, Danesi A (2020). Interplay between COVID-19, pollution, and weather features on changes in the incidence of acute coronary syndromes in early 2020. Int J Cardiol.

